# Repeated pulse feeding induces functional stability in anaerobic digestion

**DOI:** 10.1111/1751-7915.12025

**Published:** 2013-01-10

**Authors:** Jo De Vrieze, Willy Verstraete, Nico Boon

**Affiliations:** Laboratory of Microbial Ecology and Technology (LabMET), Ghent UniversityCoupure Links 653, B-9000, Gent, Belgium

## Abstract

Anaerobic digestion is an environmental key technology in the future bio-based economy. To achieve functional stability, a minimal microbial community diversity is required. This microbial community should also have a certain ‘elasticity’, i.e. the ability to rapidly adapt to suboptimal conditions or stress. In this study it was evaluated whether a higher degree of functional stability could be achieved by changing the feeding pattern, which can change the evenness, dynamics and richness of the bacterial community. The first reactor (CSTR_stable_) was fed on daily basis, whereas the second reactor (CSTR_dynamic_) was fed every 2 days. Average biogas production was 0.30 l CH_4_ l^−1^ day^−1^ in both reactors, although daily variation was up to four times higher in the CSTR_dynamic_ compared with the CSTR_stable_ during the first 50 days. Bacterial analysis revealed that this CSTR_dynamic_ had a two times higher degree of bacterial community dynamics. The CSTR_dynamic_ also appeared to be more tolerant to an organic shock load of 8 g COD l^−1^ and ammonium levels up to 8000 mg TAN l^−1^. These results suggest that the regular application of a limited pulse of organic material and/or a variation in the substrate composition might promote higher functional stability in anaerobic digestion.

## Introduction

Anaerobic digestion is a well-known and frequently used process for renewable energy production from organic waste. The European Union stated that 20% of the European energy demands should be originating from renewable energy sources by the year 2020, to which anaerobic digestion has to contribute for at least 25% (Holm-Nielsen *et al*., 2009). Anaerobic digestion will play a major role in the future bio-based economy by the conversion of low value organic products into biogas (Mata-Alvarez *et al*., 2000; Verstraete *et al*., 2005). Anaerobic digestion offers several advantages over other processes treating organic waste streams, such as the production of biogas and a substantial decrease and stabilization of the organic waste. A high loading rate, limited nutrient demands and low operational control and maintenance costs are additional advantages as well (Mata-Alvarez *et al*., 2000; Lesteur *et al*., 2011; Wijekoon *et al*., 2011).

A wide diversity in organic substrates can be converted to methane and CO_2_ by means of anaerobic digestion. Stable conversion of these diverse substrates requires functional stability, i.e. stable methane production and a certain redundancy towards stress. It is assumed that a minimal diversity in the microbial community is necessary to achieve functional stability (Briones and Raskin, 2003; Riviere *et al*., 2009). Each step in the degradation pathway of the organic compounds of the substrate is conducted by at least one microorganism. The first three steps of the anaerobic digestion system (i.e. hydrolysis, acidogenesis and acetogenesis) are carried out by bacteria, whereas archaea are responsible for the last step, i.e. methanogenesis (Gerardi, 2003). This bacteria–archaea succession normally yields an almost four times higher bacterial diversity compared with the archaeal diversity in stable anaerobic digesters (Fernandez *et al*., 1999; Briones and Raskin, 2003). Both the bacterial and archaeal diversity are however of major importance because they contribute to the stability of the digesters. A higher diversity generates the potential of multiple pathways for the degradation of a certain organic compound, hence yielding functional redundancy (Peterson *et al*., 1998; Briones and Raskin, 2003; Carballa *et al*., 2011).

It is important to indicate that microbial diversity *per se* does not implicate functional stability, rather than the ability of the microbial community to rapidly adapt to suboptimal conditions (Briones and Raskin, 2003; Dearman *et al*., 2006; Carballa *et al*., 2011). Low microbial diversity can coincide with a high functional stability, indicating that the flexibility of the community, instead of its diversity, is crucial to ensure stable operation (Haruta *et al*., 2002; Dearman *et al*., 2006). A dynamic microbial community, together with a high initial evenness are considered of vital importance to guarantee functional stability in microbial communities in general (Fernandez *et al*., 1999; Wittebolle *et al*., 2009; Boon *et al*., 2011; Carballa *et al*., 2011). The evenness, dynamics and diversity of a microbial community in the anaerobic digester depend greatly on the reactor conditions (e.g. pH, ammonium concentration and salt concentration or conductivity), feed composition (e.g. total nitrogen and organic matter content) and feeding pattern (e.g. pulse or continuous feeding) (Dearman *et al*., 2006; Krakat *et al*., 2011).

The objective of this study was to evaluate whether a higher degree of functional stability could be achieved by changing the feeding pattern which may influence the evenness, dynamics and diversity of the microbial community in anaerobic digestion. To achieve this, the effect of a difference in the feeding pattern in anaerobic digestion on (i) methane production, (ii) bacterial community evenness, dynamics and richness and (iii) tolerance of the reactor to several impairments, by means of a short-term stress test of 4 days, was investigated. The microbial resource management (MRM) approach (Marzorati *et al*., 2008; Read *et al*., 2011) was implemented to gain insight in the microbial community organization in the anaerobic digesters. The microbial community parameters range-weighted richness Rr (the amount of species), dynamics Dy (number of species that on average come to significant dominance during a defined time interval, in this case 7 days) and community organization Co (which indicates the evenness of the community) were determined, based on the bacterial denaturing gradient gel electrophoresis (DGGE) profile, and linked to the reactor performance and stress tolerance (Marzorati *et al*., 2008; Read *et al*., 2011).

## Results

### Anaerobic reactors performance

**Figure 1:**
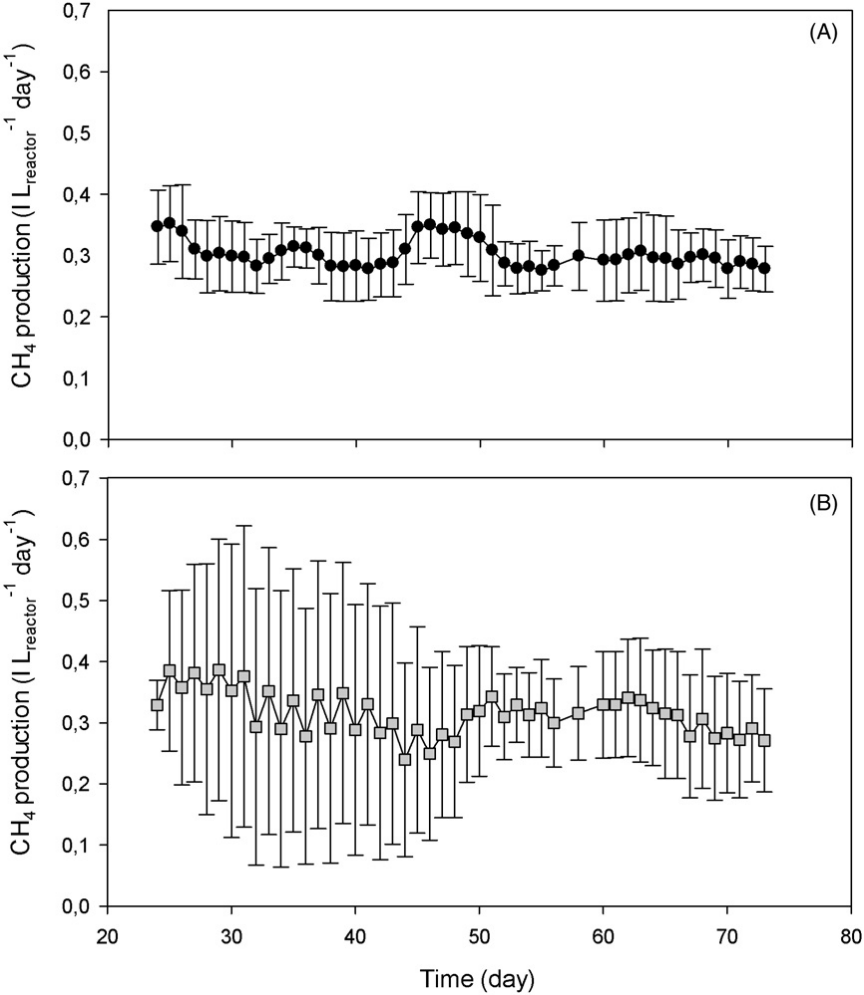
Performance of the CSTR_stable_ (•) and CSTR_dynamic_ (▪) in terms of methane production. A 7-day moving window, together with the in-window variation of the methane production has been plotted for the CSTR_stable_ (A) and the CSTR_dynamic_ (B), for each day of operation. Each value represents the average and the variation of the value on the day itself and the 6 previous days.

During the first 24 days of the experiments, both reactors were operated under similar conditions, i.e. a daily pulse loading rate of 1 g COD l^−1^ day^−1^. An average methane production of 0.31 ± 0.07 l l_reactor_^−1^ day^−1^ was achieved in both reactors (Fig. 1), which corresponded to a removal efficiency of 86.6%. On day 24, both reactors were mixed to start with the same sludge. From day 24 until day 73, both reactors were run at a different feeding pattern (i.e. daily versus every 2-day feeding). A 7-day moving window, together with the in-window variation of the methane production has been plotted for the CSTR_stable_ (Fig. 1A) and the CSTR_dynamic_ (Fig. 1B), for each day of operation. Each value represents the average and the variation of the value on the day itself and the 6 previous days. This 7-day moving window of the methane production was deviated in order to achieve an accurate comparison between methane production and the ecological parameters, which were determined every 7 days. The average methane production in the CSTR_stable_ was 0.28 ± 0.06 l l_reactor_^−1^ day^−1^ and in the CSTR_dynamic_ 0.29 ± 0.15 l l_reactor_^−1^ day^−1^. Both reactors thus demonstrated an equal average methane production, yet with elevated daily variations in the CSTR_dynamic_ compared with the CSTR_stable_. These daily variations were highest in the beginning, but slowly declined towards the end of the experiment (Fig. 1). The average COD removal efficiency was 77.8% and 81.2% over time in the CSTR_stable_ and CSTR_dynamic_ respectively.

The CSTR_stable_ and CSTR_dynamic_ had an average pH of 7.11 ± 0.07 and 7.10 ± 0.08, respectively, indicating that the average pH, as well as the variation was similar between the two reactors, although the CSTR_dynamic_ demonstrated more daily variation. Soluble COD (COD_sol_) remained below 300 mg COD l^−1^ in the CSTR_stable_, with an average value of 221 ± 47 mg COD l^−1^. This was in contrast to the CSTR_dynamic_, which demonstrated a maximum COD_sol_ concentration of 613 mg COD l^−1^ on day 73 and an average value of 347 ± 130 mg COD l^−1^. The residual VFA concentration remained below detection limit of the method (i.e. < 2 mg l^−1^) in both reactors for the entire period of the experiment. Total COD, total ammonia nitrogen (TAN), volatile solids (VS) and total solids (TS) gave similar results for both reactors (data not shown).

### Short-term stress test

**Figure 2:**
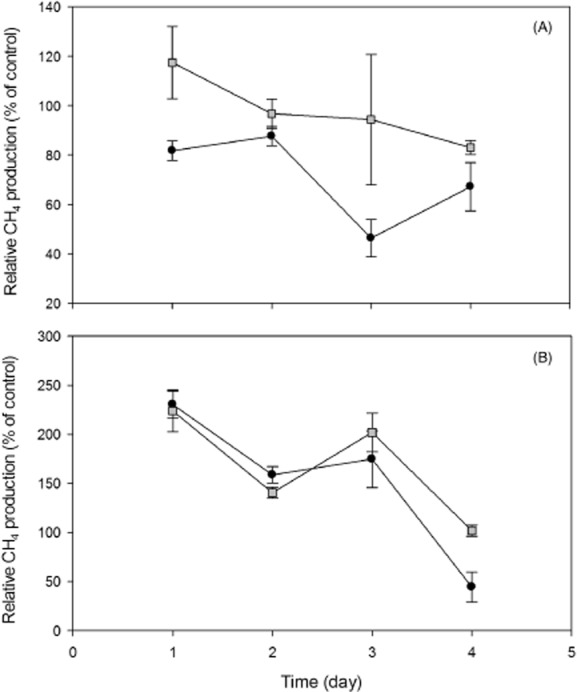
Results of the short-term stress test at the end of the experiment in terms of the tolerance of the CSTR_stable_ (•) and CSTR_dynamic_ (▪) to high concentrations of ammonium (A) and elevated organic loading rates (B). Average values of the three replicates per treatment are represented together with the values of the standard deviations as error bars.

The results of the short-term stress test at the end of the experiment in terms of the tolerance of both reactors to higher concentrations of ammonium and elevated organic loading rates are given in Fig. 2. There appears to be a substantial difference in ammonium tolerance between the two reactors, since the relative methane production (i.e. the relation between the methane production of the treatment and the control) was 10–50% higher in the CSTR_dynamic_ compared with the CSTR_stable_ (Fig. 2A), which indicates that the CSTR_dynamic_ is more tolerant to high ammonium concentrations. No remarkable differences in pH were detected.

Elevated organic loading rates seemed to have a different effect on the different reactors as well (Fig. 2B). During day 1 and 2, which corresponds to an OLR of 2 and 4 g COD l^−1^ day^−1^ respectively, no noteworthy difference in terms of methane production between both reactors could be detected. On day 3 and 4 however, during which an OLR of 6 and 8 g COD l^−1^ day^−1^ respectively was applied, the methane production was 27% higher on day 3 and even 57% higher on day 4 in the CSTR_dynamic_ compared with the CSTR_stable_. These results are also reflected in the pH, which was 6.22 ± 0.03 in the CSTR_dynamic_ and 5.04 ± 0.12 in the CSTR_stable_ on day 4, indicating severe acidification in the latter. Elevated concentrations of sulfate and the induction of acidification by means of HCl yielded no remarkable effect on methane production in and between both reactors. Methane production was not influenced by elevated sulfate concentration, i.e. there was no difference in methane production and pH between the sulfate treatment and the control. Acidification by means of HCl did decrease the pH to a value of 6.43 ± 0.01 in the CSTR_stable_ and 6.44 ± 0.03 in the CSTR_dynamic_ on day 4. This resulted in a decrease of 0.3 pH units compared with the control treatments (6.69 ± 0.05 and 6.74 ± 0.04 for the CSTR_stable_ and CSTR_dynamic_ respectively), yet methane production was only slightly affected in both reactors, i.e. a difference of 10% between the treatment and the control. This indicates that there was no remarkable difference between both reactors in terms of pH and methane production.

### Microbial community analysis

**Figure 3:**
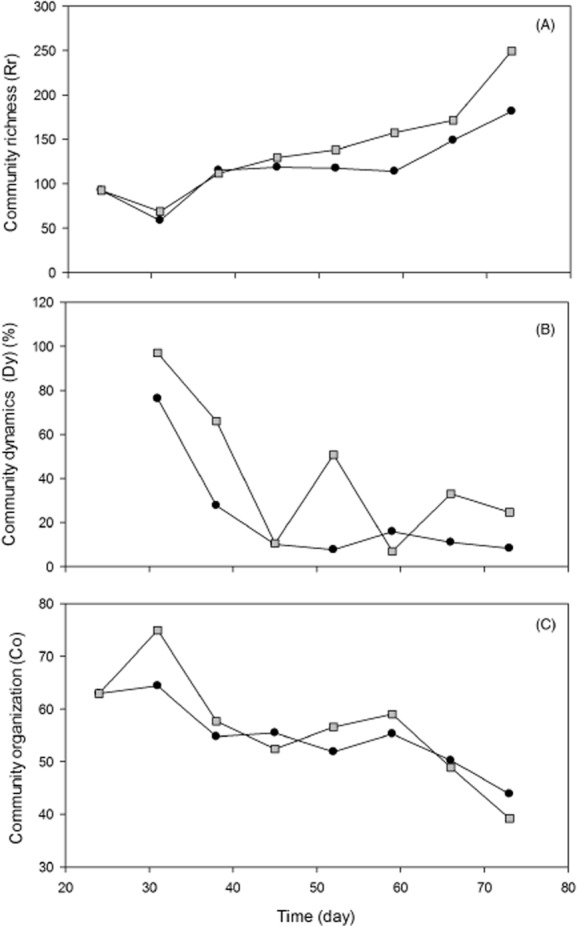
Ecological parameters range-weighted richness (A), dynamics (B) and community organization (C) of the bacterial communities in the CSTR_stable_ (•) and CSTR_dynamic_ (▪).

Figure 3 represents the ecological parameters range-weighted richness (Rr), dynamics (Dy) and community organization (Co) of the bacterial communities in the CSTR_stable_ and CSTR_dynamic_. These results are based on the DGGE profile of the bacterial community in both reactors, which is represented in Fig. S1.

Bacterial diversity was estimated by the Rr value. Both reactors started with an equal Rr value of 93 on day 24. Further operation of the two reactors in a different feeding pattern led the Rr to rise to higher levels in the CSTR_dynamic_ compared with the CSTR_stable_, although both reactors exhibited a higher Rr value at the end of the experiment (Fig. 3A). The Rr values of the CSTR_dynamic_ and the CSTR_stable_ were 250 and 182 respectively. Also the average Rr value was higher for the CSTR_dynamic_ in comparison with the CSTR_stable_, i.e. 140 ± 55 compared with 119 ± 36.

The bacterial community dynamics were evaluated using the Dy coefficient. Both reactors demonstrated very high changes in bacterial community dynamics, i.e. 76% community change after the first 7 days for the CSTR_stable_ and 97% for the CSTR_dynamic_ (day 31) following the introduction of the different feeding pattern (Fig. 3B). The 7-day community change dropped to a value < 16% within 21 days after the change of the feeding pattern in the CSTR_stable_. The CSTR_dynamic_ on the other hand not only demonstrated 7-day changes up to 50%, there was a substantial variation in the 7-day change pattern, i.e. the 7-day evolution of the dynamics was more variable, as well, which was in contrast to the CSTR_stable_.

The bacterial community evenness was reflected by means of the Co coefficient. A lower value of Co corresponds to a more even community. The two reactors each started from a higher value of Co, that is 63, and evolved towards a lower Co value at the end of the experiment, i.e. 44 for the CSTR_stable_ and 39 for the CSTR_dynamic_ (Fig. 3C). Both reactors thus evolved towards a more even community.

The qPCR results of the *Methanobacteriales*, *Methanomicrobiales*, *Methanosarcinaceae* and *Methanosaetaceae* revealed that there is no difference between the CSTR_stable_ and CSTR_dynamic_. The *Methanosaetaceae* are the dominating methanogens and remained constant throughout the entire experiment, with on average 2.2 × 10^10^ ± 1.7 × 10^9^ and 2.3 × 10^10^ ± 2.1 × 10^9^ copies g^−1^ sludge in the CSTR_stable_ and CSTR_dynamic_ respectively. The *Methanobacteriales* showed a slight increase from 3.8 × 10^8^ ± 2.5 × 10^7^ in both reactors on day 24 to 2.2 × 10^9^ ± 1.2 × 10^8^ and 2.7 × 10^9^ ± 2.4 × 10^8^ copies g^−1^ sludge on day 73 in the CSTR_stable_ and CSTR_dynamic_ respectively. The *Methanomicrobiales* showed similar values compared with the *Methanobacteriales*, yet copy numbers remained stable in both reactors in the entire experiment with average values of 1.4 × 10^9^ ± 1.3 × 10^8^ and 1.1 × 10^9^ ± 1.4 × 10^8^ copies g^−1^ sludge in the CSTR_stable_ and CSTR_dynamic_ respectively. *Methanosarcinaceae* copy numbers also remained stable and similar in the CSTR_stable_ and CSTR_dynamic_ with average values of 2.1 × 10^6^ ± 2.7 × 10^5^ and 1.9 × 10^6^ ± 3.5 × 10^5^ copies g^−1^ sludge.

### Correlations between methane production variation and the bacterial community

A moving window value of the methane production has been determined of the 7 days preceding each microbial community sampling time point (i.e. every 7 days). In order to correlate methane production variation to the ecological parameters Rr, Dy and Co, the variation of this 7-day moving window methane production was determined. The correlations between the ecological parameters and the moving window methane production variation were subsequently determined and are shown in Table 1. There was a significant negative correlation (*P* < 0.05) between the bacterial community richness and organization in both reactors and there also was a significant positive correlation (*P* < 0.05) between the bacterial community organization and the in-window variation of methane production in the CSTR_stable_ only (Table 1).

**Table 1:** Correlations between the ecological parameters Rr, Dy and Co and the moving window methane production variation, determined by means of the Spearman rank order correlation analysis, for the CSTR_stable_ and CSTR_dynamic_

CSTR_stable_		Var CSTR_stable_	Dy CSTR_stable_	Co CSTR_stable_	Rr CSTR_stable_
Var CSTR_stable_	Corr. Coeff	1.000	0.536	0.714^a^	−0.548
	Sign. level	–	0.215	0.047	0.160
Dy CSTR_stable_	Corr. Coeff	0.536	1.000	0.607	−0.714
	Sign. level	0.215	–	0.148	0.071
Co CSTR_stable_	Corr. Coeff	0.714^a^	0.607	1.000	−0.857^a^
	Sign. level	0.047	0.148	–	0.007
Rr CSTR_stable_	Corr. Coeff	−0.548	−0.714	−0.857^a^	1.000
	Sign. level	0.160	0.071	0.007	–
CSTR_dynamic_		Var CSTR_dynamic_	Dy CSTR_dynamic_	Co CSTR_dynamic_	Rr CSTR_dynamic_
Var CSTR_dynamic_	Corr. Coeff	1.000	0.536	0.048	−0.286
	Sign. level	–	0.215	0.911	0.493
Dy CSTR_dynamic_	Corr. Coeff	0.536	1.000	0.357	−0.607
	Sign. level	0.215	–	0.432	0.148
Co CSTR_dynamic_	Corr. Coeff	0.048	0.357	1.000	−0.833^a^
	Sign. level	0.911	0.432	–	0.010
Rr CSTR_dynamic_	Corr. Coeff	−0.286	−0.607	−0.833^a^	1.000
	Sign. level	0.493	0.148	0.010	–

a Correlation is significant at the 0.05 level.

## Discussion

A higher degree of functional stability was achieved by changing the feeding pattern, which altered the evenness, dynamics and diversity of the bacterial community, yet the archaeal community was not influenced. A short-term stress test revealed that the CSTR_dynamic_ was more tolerant to high levels of ammonium and high organic loading rates. The bacterial community in the CSTR_dynamic_ demonstrated a higher degree of dynamics, yet both reactors evolved towards a more even bacterial community.

Average methane production and yield remained the same in both reactors, indicating that the stronger pulse fed pattern of the CSTR_dynamic_ (fed every 2 days) did not cause an organic overloading of the reactor, i.e. no fatty acids were detected and the pH remained stable. These results are in agreement with an earlier study, in which only little difference in average biogas production was detected between an hourly and a daily fed reactor (Conklin *et al*., 2006). Daily variation in methane production was however much higher in the CSTR_dynamic_, compared with the CSTR_stable_, which is reflected in the in-window variation of the methane production of both reactors. This higher degree of variation in the CSTR_dynamic_ was also reflected in a higher degree of variation in the pH and COD_sol_ in this reactor, compared with the CSTR_stable_. These observations correspond with the ones of Conklin and colleagues (2006), who had a higher standard deviation of methane production and a higher degree of variation in pH in the daily fed reactor, compared with the hourly fed reactor.

Bacterial community analysis revealed that the Rr values reached 250 and 182 at the end of the experiment in respectively the CSTR_dynamic_ and CSTR_stable_, while in other anaerobic CSTR reactors the bacterial richness never exceeded a Rr value of 40 (Carballa *et al*., 2011; Pycke *et al*., 2011). The difference in bacterial richness is quite low and despite the fact that it diverges towards the end of the experiment, it can be stated that bacterial richness is similar in both reactors. When comparing these results to the Rr values of different microbial communities in different environments, as listed by Marzorati and colleagues (2008), it can be stated that bacterial richness was very high in the reactors in this study. This can be correlated to the diversity of the substrate, which consisted of several different organic compounds (Table 2), as the application of only one substrate to the anaerobic digester strongly reduces or limits bacterial richness (Fernandez *et al*., 1999; Delbes *et al*., 2000; Zamalloa *et al*., 2012).

**Table 2:** Composition of the synthetic feed

Component	Amount
*Carbon source*	(mg l^−1^)
Starch	18 000
Milk powder	2000
Yeast extract	200
Tryptic soy	200
*Buffer*	(mM)
KH_2_PO_4_	10
K_2_HPO_4_	10
NaHCO_3_	20
*Macronutrients*	(mg l^−1^)
NH_4_Cl	500
CaCl_2_·2H_2_O	200
MgCl_2_·6H_2_O	100
Fe_2_(SO_4_)_3_	100
*Trace elements*	(μg l^−1^)
NiSO_4_·6H_2_O	500
MnCl_2_·4H_2_O	500
FeSO_4_·7H_2_O	500
ZnSO_4_·7H_2_O	100
H_3_BO_3_	100
Na_2_MoO_4_·2H_2_O	50
CoCl_2_·6H_2_O	50
CuSO_4_·5H_2_O	5

Bacterial community dynamics in the CSTR_dynamic_ demonstrated 7-day changes up to 50%, which can be considered a high value of dynamics, when compared with other (anaerobic) ecosystems, which had an average 7-day dynamics of 25% (Marzorati *et al*., 2008; Wittebolle *et al*., 2009; Carballa *et al*., 2011; Pycke *et al*., 2011). This higher degree of dynamics is however not negatively correlated to operational stability, since the CSTR_dynamic_ produced equal levels of methane as the CSTR_stable_. This was also reflected in the studies of Fernandez and colleagues (1999), who stated that extremely dynamic communities can still maintain high functional stability and that a high degree of bacterial diversity, which is also the case in these reactors, can contribute to high levels of dynamics. This high level of dynamics in correlation with a high bacterial diversity also implies that the CSTR_dynamic_ will be able to rapidly respond to changing conditions (Dearman *et al*., 2006; Verstraete *et al*., 2007).

The bacterial community evolved towards a more even community in both reactors, which was similar in both reactors. This community organization can be considered as a measure for the degree of functional organization of the bacterial community, i.e. the higher the Co value, the more specialized the bacterial community is (Marzorati *et al*., 2008; Read *et al*., 2011). On the other hand, a very uneven community can be considered as being less resilient to changing conditions because of its high level of specialization (Wittebolle *et al*., 2009). A stable community therefore needs to contain a certain level of organization (more uneven) but also a level of functional resilience (more even), to which both community richness and dynamics can contribute (Fernandez *et al*., 1999; Marzorati *et al*., 2008; Wittebolle *et al*., 2009). The evolution of both reactors towards a more even community, compared with community at the start of the experiment, can be explained by the diversity of the substrate, requiring multiple bacterial species to degrade all compounds.

Although the ecological parameters, based on the DGGE profile, represent valuable information concerning the bacterial community, caution should be taken with the interpretation of the data, since the DGGE method has some well-known limitations. The number and abundance of bacterial species in the anaerobic digester is not exactly reflected by the number and intensity of the bands (Boon *et al*., 2002). One bacterial species may demonstrate more than one band, one band may represent multiple species and species which have an abundance < 1% cannot be visualized by means of DGGE (Boon *et al*., 2002), thus only dominant species were taken into account, which was the goal of this research. When interpreting these ecological parameters deducted from any molecular analysis, one should be aware of the limitations of the techniques used.

Real-time PCR results demonstrated that there was no difference in methanogenic community composition between the two reactors and also that there was only a slight increase in *Methanobacteriales* copy numbers, the other groups remaining constant. This is in contrast to the bacterial community, which showed a substantial change throughout the experiments, with different levels of dynamics in the two reactors. The presence of the different methanogenic groups however demonstrates that both acetoclastic and hydrogenotrophic methanogenesis took place in both reactors, yet the dominance of the *Methanosaetaceae* in the two reactors assigns acetoclastic methanogenesis as the dominant pathway. This is however to be expected, since residual VFA concentrations were below detection limit at all times in the two reactors. Since *Methanosaeta* sp*.* show a high affinity for acetate compared with *Methanosarcina* sp*.*, they tend to be the dominant acetoclastic methanogens at low acetate concentrations, which immediately also explains the low *Methanosarcinaceae* copy numbers (De Vrieze *et al*., 2012). It was shown in the study of Conklin and colleagues (2006) that there was a clear shift from a *Methanosaeta* to a *Methanosarcina* dominated methanogenic community at higher interval feeding, which was not the case in this research because of the very low residual acetate concentrations.

Spearman rank order correlation coefficients between the ecological parameters and the in-window methane production variation are represented in Table 1. The strong negative correlation coefficient between the bacterial community richness and organization in both reactors indicated that a higher degree of bacterial community evenness can be directly correlated to a higher bacterial diversity, a similar result which was obtained in the research of Carballa and colleagues (2011). Unfortunately, our results could not be related to the in-window methane production variation. However these results, together with the results of Carballa and colleagues (2011) indicated that bacterial richness in anaerobic digestion can be predicted by the bacterial community organization and vice versa, which does not particularly seem to be the case in other bacterial ecosystems. It can be deducted from the positive correlation between community organization and operational variation in the CSTR_stable_ that a bacterial community with high evenness (low Co value) causes limited process variation, whereas a community with only a few dominant species (high Co value) leads to more process variation. This might attribute an extra dimension to the findings of Wittebolle and colleagues (2009), who reported that initial evenness contributes to functional stability. Community unevenness may lead to operational variation under normal or optimal conditions and when the community evolves towards a more even community, process variation declines.

The higher tolerance of the CSTR_dynamic_ to higher levels of ammonium and high organic loading rates is in agreement to the study of Conklin and colleagues (2006), which demonstrated that daily feeding compared with hourly feeding in anaerobic digestion led to a higher tolerance to organic overloading. Yet, the latter authors did not detect a higher tolerance to ammonium stress and no relation with the bacterial community was established. The elevated tolerance of the CSTR_dynamic_ to ammonium stress can be related to its more variable methane production profile. Indeed, a higher resistance to ammonium stress can be induced by means of a pulse feeding pattern and a subsequent higher degree of methane production variation can be a sign of the latter. This elevated ammonium tolerance can be correlated to the enhanced levels of dynamics of the bacterial community as well, which is also shown in the study of Fernandez and colleagues (1999). The latter study demonstrated that a more flexible microbial community is correlated to a higher degree of stability when exposed to a shock load of glucose, thus connecting process stability to bacterial community dynamics. Our study demonstrated that the elevated resistance to impairments can be reflected, not only in community dynamics, but in other ecological parameters as well. That is, higher community diversity and higher degree of dynamics in the bacterial community can thus be correlated to more process stability at suboptimal conditions. This supports the hypothesis of Verstraete and colleagues (2007) that stable processes do not host a stable climax community but that there is always a certain degree of diversity and dynamics required to ensure continuous stable operation. A higher degree of process stability, i.e. higher tolerance to common forms of stress, can thus be achieved by introducing a pulse feeding pattern in anaerobic digestion.

In conclusion this study demonstrated that stable operation can be maintained in anaerobic digestion when stronger pulse feeding patterns are applied, although at the cost of more daily operational variation. A pulse feeding pattern leads to a higher degree of bacterial dynamics, which can, together with a higher bacterial diversity, be correlated to a higher tolerance to high levels of ammonium and organic overloading in anaerobic digestion. The methanogenic community remained stable in both reactors, with a clear dominance of the *Methanosaetaceae*. These results call for the regular application of a limited pulse of organic material and/or a variation in the substrate to obtain a higher degree of functional stability in the anaerobic digester. Molecular fingerprinting techniques, e.g. DGGE, thus provide valuable information concerning the microbial community in the anaerobic digester. Further research concerning the role of initial evenness of the bacterial and archaeal community and its evolution in terms of process stability will provide more valuable information to further steer anaerobic digestion. Also the application of next-generation sequencing techniques might provide interesting information concerning the identity of the dominant species and the role of species present at low abundance.

## Experimental procedures

### Experimental set-up and operation

Two anaerobic lab-scale continuously stirred tank reactors (CSTR), each with a total volume of 10 l and a working volume of 8 l, were operated for 73 days under mesophilic conditions, i.e. 34°C, at a hydraulic retention time (HRT) of 20 days. An operational volume of 8 l was chosen for the reactors, since these reactors are quite reproducible, as indicated in earlier preliminary research (data not shown) and other papers by Wittebolle and colleagues (2008), Carballa and colleagues (2011) and Zamalloa and colleagues (2012). The reactors were inoculated with mesophilic sludge, which originated from a domestic wastewater treatment plant (Ossemeersen, Belgium). This sludge was diluted with tap water until a volatile suspended solids (VSS) concentration of 10 g VSS l^−1^ was obtained. The two reactors were both subjected to a daily pulse loading rate of 1 g COD l^−1^ day^−1^ during the first 24 days of the experiment. After 24 days, this daily feeding pattern was continued in reactor one (CSTR_stable_), whereas the second reactor (CSTR_dynamic_) was fed every 2 days with the same average loading rate of 1 g COD l^−1^ day^−1^. The composition of the synthetic feed used, which is based on the SYNTHES feed, is given in Table 2 (Aiyuk and Verstraete, 2004). This SYNTHES feed is a synthetic raw domestic sewage suitable for anaerobic digestion and was developed in order to apply a feed with constant stable characteristics (Aiyuk and Verstraete, 2004). This SYNTHES feed contains all components necessary to ensure stable operation of the microbial community in anaerobic digestion.

The pH of both reactors was monitored and adjusted on daily basis to a value of 7.2 with a NaOH solution of 2 M. The biogas production and the percentage of methane in the biogas was measured on daily basis and reported at STP (standard temperature and pressure) conditions. Total biogas production was monitored by means of a gas meter. Effluent samples were taken trice a week for volatile fatty acids (VFA) analysis and once a week for soluble COD (COD_sol_) and total ammonia nitrogen (TAN). From day 24 on, a sample of the anaerobic biomass was taken every week to examine the bacterial community. These samples were subsequently stored at −20°C until DNA extraction was performed.

### Short-term stress test

The short-term stress test at the end of the experiment, i.e. after 73 days, was implemented to estimate the tolerance of both reactors to high concentrations of TAN and sulfate, low values of pH and high organic loading rates. Several subsamples were taken of the two main reactors on day 73 and all treatments were performed on three samples, which can be considered biological replicates, from each reactor. Ammonium was applied as NH_4_Cl, sulfate as Na_2_SO_4_ and the pH was lowered with a 2 M HCl solution. The same feed as during operation of the main experiment was used for both the normal feeding and the high OLR treatment. All treatments for both reactors received a daily feeding of 1 g COD l^−1^ day^−1^, with the exception of the high organic loading rate treatment in which the OLR was raised every day. The set-up of the stress test is given in Table 3. The test was carried out in airtight penicillin bottles with a volume of 100 ml, which contained 50 ml of sludge from the CSTR_stable_ or CSTR_dynamic_ during a period of 4 days. Both biogas production and composition and pH were measured on daily basis. Feeding was performed and samples were taken by means of a syringe to keep the bottles air-tight. Gas production was monitored by means of a gas syringe.

**Table 3:** Short-term stress test set-up

Stressor	Day 1	Day 2	Day 3	Day 4
Control	–	–	–	–
Ammonium (mg TAN l^−1^)	1000	2000	4000	6000
Sulfate (mg l^−1^)	500	1000	2000	4000
High OLR (g COD l^−1^ day^−1^)^a^	2	4	6	8
Acidification with HCl (mmol l^−1^)	2	6	12	18
Acidification with HCl (final pH)	7.27 ± 0.05	6.97 ± 0.06	6.72 ± 0.04	6.44 ± 0.02

a In every treatment, the OLR was 1 g COD l^−1^ day^−1^, except for the high organic loading rate treatment in which the OLR was raised every day, as presented in the table.

The values presented for ammonium, sulfate and acidification are final concentrations in the reactor (*n* = 3).

### Microbial community analysis

Total DNA was extracted from the sludge samples and subsequently purified according to the method of Boon and colleagues (2000). DGGE on the total bacterial community was performed following the PCR protocol of Boon and colleagues (2002), using the total bacterial primers P338f-GC and P518r (Muyzer *et al*., 1993). The PCR was run with a 2720 thermal cycler (Applied Biosystems). The presence and size of the PCR product was verified on a 1% agarose gel. An INGENY phorU2X2 DGGE system (Goes, the Netherlands) was subsequently used to run an 8% (w/v) polyacrylamide DGGE gel with a denaturing gradient ranging from 45% to 60%, consistent with the protocol of Boon and colleagues (2002). The obtained DGGE gel was processed using the Bionumerics software 5.1 (Applied Maths, Kortrijk, Belgium). Only bands with an intensity higher than 1% were considered. The DGGE results were used to estimate the theoretical ecological parameters range-weighted richness (Rr), dynamics (Dy) and community organization (Co), as stated above, of the bacterial communities in both reactors (Marzorati *et al*., 2008; Read *et al*., 2011). The Rr values were determined based on the number of bands in the DGGE pattern and the percentage of the denaturing gradient between the first and the last band of the pattern, as described by Marzorati and colleagues (2008). A matrix of similarities between the densiometric curves of the band patterns was calculated on the basis of the Pearson product–moment correlation coefficient, from which the Dy values were deducted (Marzorati *et al*., 2008). The Co value was determined based on the number and the intensity of the bands in the DGGE pattern. This value is deducted from the Gini coefficient, which describes a specific degree of evenness, by means of a measurement of the normalized area between a given Pareto-Lorenze curve and the perfect evenness line. The higher the Co value, the more uneven the community is (Marzorati *et al*., 2008; Wittebolle *et al*., 2009).

Real-time PCR (qPCR) was performed on a StepOnePlus™ Real-Time PCR System (Applied Biosystems, Carlsbad, CA). Triplicate samples of a 10-to 100-fold dilution of the DNA samples were analysed for *Methanobacteriales*, *Methanomicrobiales*, *Methanosarcinaceae* and *Methanosaetaceae*. The primer sets were previously described by Yu and colleagues (2009). A reaction mixture of 20 μl was prepared by means of the GoTaq qPCR Master Mix (Promega, Madison, WI) containing 10 μl of GoTaq® qPCR Master Mix, 3.5 μl of nuclease-free water and 0.75 μl of each primer (final concentration of 375 nM) and 5 μl of template DNA. The qPCR program was performed in a two-step thermal cycling procedure for all four groups which consisted of a pre-denaturation step of 10 min at 94°C, followed by 40 cycles of 10 s at 94°C and 1 min at 60°C. The qPCR data were represented as copies per gram of sludge.

### Analytical techniques

Total suspended solids (TSS), VSS, TAN, total COD (COD_tot_) and COD_sol_ were determined according to Standard Methods (Greenberg *et al*., 1992). The VFA were extracted with diethyl ether and measured in a GC-2014 gas chromatograph (Shimadzu, ‘s-Hertogenbosch, the Netherlands), which was equipped with a capillary fatty acid-free EC-1000 Econo-Cap column (dimensions: 25 mm × 0.53 mm, film thickness 1.2 mM; Alltech, Laarne, Belgium), a split injector and a flame ionization detector. The lower detection limit for VFA analysis was 2 mg l^−1^. Biogas composition was analysed with a Compact GC (Global Analyser Solutions, Breda, the Netherlands), which was equipped with a Porabond precolumn and a Molsieve SA column. Concentrations of CH_4_, CO_2_ and H_2_ were determined by means of a thermal conductivity detector with a lower detection limit of 1 ppmv for each gas component. The pH was measured with a C532 pH meter (Consort, Turnhout, Belgium).

### Statistical analysis

Correlation coefficients between the ecological parameters Rr, Dy and Co and the variation of the 7-day moving window average methane production were determined by means of the two-tailed Spearman's Rank Order Correlation test, for which the statistical software SPSS (IBM SPSS Statistics, Version 20, IBM Corp., Armonk, New York, USA) was used. This software was applied to estimate whether there was a significant linear correlation. A statistical significance level of 5% was applied.

## Supporting information

Fig S1 DGGE profile of the bacterial community in the CSTR_stable_ (S1–S7) and CSTR_dynamic_ (D1–D7) from day 31 to day 73 of the experiment. Both reactors started with the same sludge inoculum on day 24 (I). The markers are given by the letter M.
