# Supplementary figures and images for: Repeated pulse feeding induces functional stability in anaerobic digestion
Source: Microb Biotechnol. 2013 Jan 10;6(4):414–24. doi: 10.1111/1751-7915.12025 (PMC3917476; doi:10.1111/1751-7915.12025)

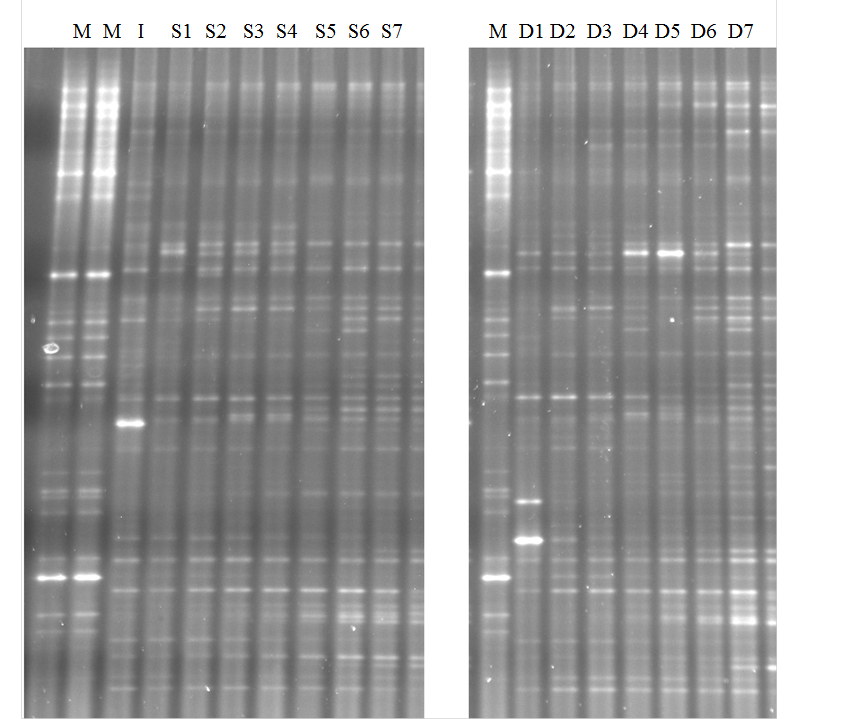

Supplement: Fig S1 — DGGE profile of the bacterial community in the CSTRstable (S1–S7) and CSTRdynamic (D1–D7) from day 31 to day 73 of the experiment. Both reactors started with the same sludge inoculum on day 24 (I). The markers are given by the letter M. [file mbt20006-0414-sd1.tif]
